# High Purity and Low Molecular Weight Lignin Nano-Particles Extracted from Acid-Assisted MIBK Pretreatment

**DOI:** 10.3390/polym12020378

**Published:** 2020-02-08

**Authors:** Qilin Zhang, Haichao Li, Zongwei Guo, Feng Xu

**Affiliations:** 1 Beijing Key Laboratory of Lignocellulosic Chemistry, Beijing Forestry University, Beijing 100083, China; zql91824@bjfu.edu.cn (Q.Z.); lihaichao96@bjfu.edu.cn (H.L.); guozongwei@bjfu.edu.cn (Z.G.); 2 MOE Key Laboratory of Wooden Material Science and Application, Beijing Forestry University, Beijing 100083, China

**Keywords:** lignin nano-particles, organosolv pretreatment, *Eucalyptus* wood, biomass, biorefinery

## Abstract

A simple and economical biorefinery method, organosolv methyl isobutyl ketone (MIBK) pretreatment assisted by Lewis acid ferric trichloride hydrolysis, was proposed for fractionating the lignin from extractive-free *Eucalyptus* powder at the nanoscale, accompanied by another product furfural, derived from hemicellulose. Under the conditions (180 °C, 1 h) optimized based on the best yield of furfural, 40.13% of the acid-insoluble lignin (AIL) could be obtained with a high purity of 100%, a low molecular weight of 767 (*M*_n_) and improved thermostability. The extracted lignin was characterized by its chemical structure, thermostability, homogeneity, molecular weight, and morphology as compared with milled wood lignin (MWL). The results showed significant variations in chemical structures of the extracted lignin during the pretreatment. Specifically, the aryl ether linkage and phenylcoumarans were broken severely while the resinols were more resistant. The G-type lignin was more sensitive to degradation than the S-type, and after the pretreatment, H-type lignin was formed, indicating the occurrence of a demethoxylation reaction at high temperature. Moreover, the lignin nano-particles were identified visually by AFM and TEM images. The dynamic light scattering (DLS) showed that the average diameter of the measured samples was 131.8 nm, with the polydispersity index (PDI) of 0.149. The MIBK-lignin nano-particles prepared in our laboratory exhibit high potentials in producing high functional and valuable materials for the application in wide fields.

## 1. Introduction

Due to abundant greenhouse gas and severe environmental problems caused by fossil fuels and the limited non-renewable energy, the pathway of valorizing the renewable lignocellulosic biomass for materials, chemicals, and biofuels has attracted many interests in recent decades [1,2]. Lignocellulose is mainly composed of cellulose, hemicellulose, and lignin. Specifically, cellulose and hemicellulose are classes of polymers containing one or several monosaccharide units. Especially for cellulose, a component of biomass in yielding specialty chemicals and functional materials as a result of the simple chemical composition and crystalline structure [3]. However, cellulose is well protected by the surrounding biomass recalcitrance, a barrier structured by hemicellulose and lignin. Lignin is the main factor hindering the cellulose valorization; however, on the other hand, it is also one of the few natural large-scale sources of aromatic compounds [4]. Three types of methoxylated phenylpropanoid units including *p*-hydroxyphenyl (H), guaiacyl (G), and syringyl (S) coupled by aryl ether (α–*O*–4, β–*O*–4, and 4–*O*–5) or carbon-carbon linkage (β–β, β–1, β–5, and 5–5) construct such complex lignin architecture [5,6]. Abundant aromatic functional groups make it useful for renewable chemicals or functional materials [7,8,9]. Therefore, an effective pretreatment to fractionate lignin, cellulose, and even hemicellulose from lignocellulosic biomass will facilitate economic biorefinery.

Some common pretreatments have been proved effective in breaking the biomass recalcitrance using sulfuric acid, sodium hydroxide, organosolv, ionic liquids, and deep eutectic solvents, etc. [10,11,12,13]. Due to the diseconomy and pollution caused by the effluent from acid and alkali pretreatment, organosolv pretreatment, based on the principle of dissolving lignin by certain organic solvents, presents a promising alternative [14,15]. EtOH and MeOH are the common solvents applied in organosolv pretreatment for economic reasons, although a number of organosolv have been developed, including ketones, esters, phenols, and high boiling alcohols [15,16]. Generally, the organosolv pretreatments incubate biomass with plenty of organic or aqueous-organic solvents at 150–200 °C catalyzed by acids such as sulfuric acid, formic acid, etc. [15,17,18] El Hage et al. [19] studied the lignin extraction by ethanol organosolv pretreatment at 190 °C for 60 min, and the results showed that the β–*O*–4 linkages and ester bonds were mainly cleaved; however, the core of the lignin structure did not show any significant change. Although the lignin extracted by organosolv pretreatments generally shows a number of advantages, including high purity, low molecular weight, and sulfur-free process [20]; however, the poor yield of the lignin fraction and the less effect on hemicellulose still block the downstream biorefinery process. A two-step procedure combined with dilute-acid soakage with an aqueous-ethanol organosolv treatment was proposed by Brosse et al. [18]. The yield of ethanol organosolv lignin was improved remarkably, reaching 71%. Moreover, 73% of xylose from the raw material was recovered, which indicated that the removal of hemicellulose also potentially benefited the organosolv treatment for lignin. To simplify such a two-step treatment, the aqueous-ethanol organosolv was modified by adding the hydrophobic organosolv, methyl isobutyl ketone (MIBK), resulting in the ternary mixture divided into two phases. Hence, the hemicellulose degradation occurred in the aqueous phase, while the organic solvent dissolved the lignin constantly [21,22]. Bozell et al. [16] proposed a new organosolv pretreatment process for fractionating lignocellulosic material into cellulose, hemicellulose, and lignin using a ternary mixture of MIBK, ethanol, and water with a sulfuric acid concentration of more than 0.1 M. The results showed that more than 95% of the lignin and hemicellulose was removed from the raw materials, giving a high content of cellulose, approaching 98% after clean fractionation process. However, it is still unsatisfactory in the recovery of the hemicellulose and lignin. Although current studies on pretreatment systems for removing lignin or breaking the biomass recalcitrance has focused on the ionic liquids (ILs) [23,24,25,26,27] and deep eutectic solvents (DESs) pretreatments [28,29,30,31,32], the challenge of the cellulose valorization without compromises of lignin and hemicellulose fractions is still unresolved. 

In this work, a simple and economical pretreatment method is proposed for good valorization of both lignin and hemicellulose. The organosolv pretreatment on extractives-free *Eucalyptus* sample was modified by adding the Lewis acid, ferric trichloride solution, aiming at good degradation of the hemicellulose in the aqueous phase, simultaneously facilitating the lignin dissolution by MIBK. Additionally, this biphasic system is also advantageous in producing furfural, a valuable platform chemical derived from hemicellulose. Hence, the conditions were optimized based on the maximum yield of furfural to preferentially make full use of the hemicellulose and the lignin was isolated from the MIBK phase. To investigate the chemical structure conversions of the extracted MIBK-lignin during the pretreatment, milled wood lignin (MWL) was prepared for a control, and characterization methods such as solution-state two-dimensional heteronuclear single-quantum coherence NMR (2DHSQC NMR) spectroscopy, gel permeation chromatography (GPC), atomic force microscopy (AFM), and Fourier transform infrared spectroscopy (FTIR), etc. were applied. This pretreatment method provides a potential for both the lignin and hemicellulose valorization.

## 2. Materials and Methods

### 2.1. Materials

Methyl isobutyl ketone (MIBK) was purchased from Macklin (Shanghai, China). Iron chloride hexahydrate was provided by Aladdin (Shanghai, China). All the chemicals were of analytical grade and used without further purification. *Eucalyptus* slices were collected by Sunpaper Industry Joint-Stock Co., Ltd. (Jining, China). The *Eucalyptus* slices were ground by an FZ120 plant shredder (Truelab, Shanghai, China). Then the *Eucalyptus* powder was extracted with toluene/ethanol (2:1, *v/v*) in a Blst-250SQ Soxhlet apparatus (Bilon, Shanghai, China) for 12 h, and air-dried for 24 h for further use. 

### 2.2. Isolation of MIBK-Lignin

The isolation of MIBK-lignin was carried out in a 304 stainless steel batch reactor with a 50 mL capacity polyphenylene lining (Zhengzhou Boke instrument equipment Co., Ltd., Zhengzhou China). The *Eucalyptus* sample (0.4 g) was soaked in 4 mL, and 0.06 mol/L of FeCl_3_ solution and then was added 16 mL of MIBK. The reactor was heated to 180 °C with an average heating rate of 2 °C/min and kept for 1 h. Then the reaction was stopped by the tap water to cool down to the room temperature immediately. The upper phase MIBK was collected, followed by the rotary evaporation. The ethanol (40 mL) was added to the concentrated lignin MIBK solution for better dispersion, then dropped in distilled water (500 mL) to precipitate the lignin. Finally, the MIBK-lignin sample was obtained by filtration, using a filter membrane with 0.22 μm pore diameters and then freeze-dried for 48 h. 

### 2.3. Isolation of Milled Wood Lignin (MWL)

The MWL from *Eucalyptus* was obtained by ball-milling for 12 h with a speed of 450 rpm, then extracted with 1,4-dioxane for 48 h, according to the modified Björkman’s method [5]. 

### 2.4. Chemical Composition of MIBK-Lignin

The chemical composition of MIBK-lignin was analyzed according to the National Renewable Energy Laboratory (NREL) methods [33]. The yield and purity of MIBK-lignin were calculated based on the acid-insoluble lignin in the biomass. 

### 2.5. 2D-HSQC NMR Analysis

The lignin sample (40 mg) was dissolved in 0.5 mL of DMSO-*d*_6_ and analyzed by NMR (Bruker Advance 400 MHz instrument, Karlsruhe, Germany) with a 5 mm gradient probe at room temperature.

### 2.6. FT-IR Spectroscopy Analysis

The lignin sample was analyzed by Tensor II FT-IR spectroscopy (Bruker, Karlsruhe, Germany) at room temperature. The lignin sample was pelleted with KBr. The number of scans were 128 for each sample, and the distinguishability was 4 cm^−1^ over the range of 3800 to 800 cm^−1^ in the transmission mode.

### 2.7. Gel Permeation Chromatograph (GPC) Analysis

The weight-average (*M*_w_) and number-average (*M*_n_) of lignin sample were detected by Agilent 1200 gel permeation chromatography (Agilent, Santa Clara, CA, USA) equipped with a refraction index detector (RID) and a PL-gel Mixed-B column (300 mm × 7.5 mm). The mobile phase was tetrahydrofuran (THF) with the flow rate of 1 mL/min. The column and detector temperature were set as 40 °C. 

### 2.8. Thermogravimetric Analysis

The thermogravimetric analysis (TGA) of the lignin sample was determined by the thermal analyzer (SDT Q600, TA Instrument, New Castle, DE, USA). The sample (5 mg) was placed in an Al_2_O_3_ crucible (70 μL) heating rate of 10 °C/min from 25 to 550 °C under a nitrogen atmosphere with a flow rate of 50 mL/min.

### 2.9. Atomic Force Microscopy (AFM) Images

The acquired MIBK-lignin was dispersed in deionized water at a concentration of 0.2 g/L followed by ten-minute ultrasound treatment. The upper suspension liquid was dropped on the clean mica surface then air-dried. The morphology of the MIBK-lignin sample was observed by Atomic force microscopy (Bruker Multimode 8 instrument, Karlsruhe, Germany).

### 2.10. Transmission Electron Microscopy (TEM) Images

The acquired MIBK-lignin was dispersed in deionized water with ten-minute ultrasound treatment. The upper suspension liquid was dropped on the carbon-coated copper grid and air-dried overnight. The sample was observed by JEM-1010 TEM (JEOL, Tokyo, Japan). 

### 2.11. Dynamic Light Scattering (DLS) Analysis

The lignin nano-particle diameters were analyzed by Nano-ZS90 (Malvern, UK). The sample was dispersed in deionized water with ten-minute ultrasound treatment, and the upper suspension liquid was collected for measurements. 

## 3. Results and Discussion

### 3.1. Purity and Molecular Weight Distributions

MIBK-lignin was compared with MWL in yield, purity, and molecular weight illustrated in Table 1. Due to the mild extraction conditions (room temperature), MWL yield is commonly below 20% based on the lignin existed in the original biomass, and in this work, the MWL yield from *Eucalyptus* was 8.53%. However, our MIBK-lignin was isolated under pretty high temperature (180 °C). Simultaneously, the hemicellulose was degraded significantly under a weakly acidic system, which facilitated the lignin extraction. Moreover, the organic solvent MIBK can prevent the polysaccharide from extracting into the organic phase due to poor solubility. Hence, the MIBK-lignin was obtained with a competitive yield (40.31%) and a unique high purity (100%).

The weight-average (*M*_w_), number-average (*M*_n_) molecular weights, and the polydispersity index (PDI, *M*_w_/*M*_n_) of MIBK-lignin and MWL were also analyzed by gel permeation chromatography (GPC). According to Table 1, a significant decrease in both *M*_w_ and *M*_n_ of MIBK-lignin compared with MWL was presented. MWL is considered as the alternate to the native lignin; hence, it tends to remain in the form of a larger molecular weight (1940 g/mol of *M*_n_ and 3417 g/mol of *M*_w_) due to little damage to its chemical structures. After the pretreatment, however, the MIBK-lignin was decreased to 767 and 1227 in *M*_n_ and *M*_w,_ respectively, indicating the linkages between lignin units, generally, the aryl ether linkages were broken. According to the previous results [21,26,27,28] showed in Table 2, the molecular weights of the extracted lignin from other systems and deep eutectic solvents (DESs), etc., still remained in several thousand grams per mole, which may ascribe to the condensation of the small lignin fractions by forming excess carbon-carbon bonds. This indicated, in some way, that less condensation occurred during the pretreatment of MIBK-lignin, which is considered as the result of the weak acidic conditions and the protection of the MIBK phase. The PDI describes the homogeneity of the lignin samples. Obviously, the PDI of MIBK-lignin (1.60) is smaller than that of MWL (1.76), demonstrating that the more homogeneous lignin was produced after the pretreatment. These characters indicate that the MIBK-lignin has great potential in high-value applications of lignin valorization. 

### 3.2. Thermal Analysis

**Figure 1:**
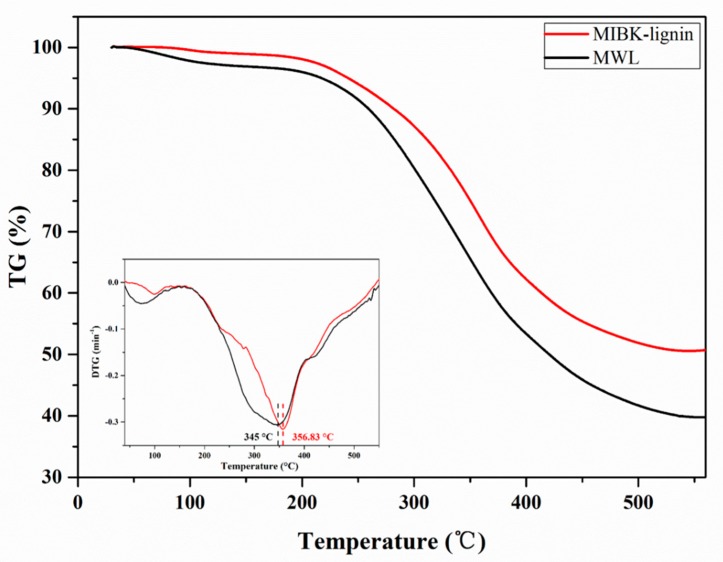
TGA and DTG curves of lignin fractions.

The MIBK-lignin was compared with MWL in thermal properties by using a thermogravimetric (TGA) analyzer. The thermal analysis was carried out in the temperature range of 30 to 550 °C, and the results are shown in Figure 1. As shown, the lignin degradation was recorded in two stages. The initial decomposition was done before the temperature approaches to 160 °C. The evaporation of water and the micromolecular lignin fraction are the main factors of the weight loss at this stage. From 160 to 550 °C was the main pyrolysis of the lignin. At this stage, both the MIBK-lignin and MWL showed a remarkable decrease in weight. From the DTG curves, it can be clearly seen that the degradation temperature ranges (from 161 to 550 °C) of the two classes of lignin were similar. However, the MIBK-lignin showed the maximum weight-loss rate at 356.83 °C, which is slightly superior to the MWL (345 °C). It indicates that the lignin was improved in thermostability after pretreatment and extraction. At 550 °C, the residual mass of MIBK-lignin was 50.7%, higher than that of the MWL (39.8%). At this stage, the demethoxylation and re-condensation of volatile products mainly caused the formation of char, resulting in the weight stabilization. Based on the above-mentioned results, the MIBK-lignin obtained in this pretreatment endowed a better thermostability. 

### 3.3. FT-IR Analysis

**Figure 2:**
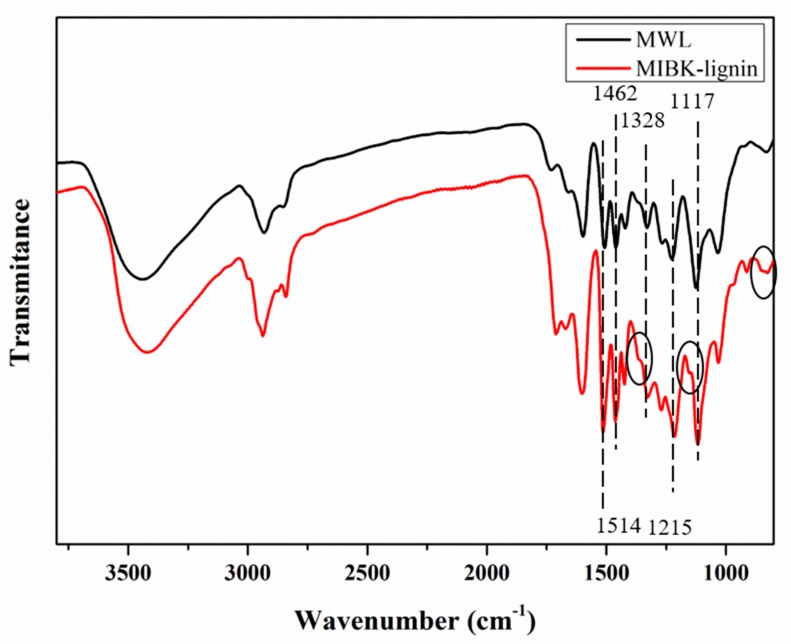
FT-IR spectra of lignin samples.

The FT-IR spectra of water generated MIBK-lignin were compared with MWL. As shown in Figure 2, the peak at 1514 cm^−1^ and 1426 cm^−1^ were typically related to aromatic skeletal vibrations indicating the spectra corresponded to lignin. The broad absorbance peak at 3421 cm^−1^ was attributed to the stretching vibrational bands of a hydroxyl group from alcohol and phenol. The increase in the intensity of double peaks of MIBK-lignin at 2936 cm^−1^ and 2840 cm^−1^ were C–H stretching in methyl and methylene groups respectively and possibly was the result of the elimination of functional groups on the side chain during the pretreatment. According to the previous work [38], the region of lower wavenumber, particularly the fingerprint region, can be used to distinguish lignin classification. From the MWL spectra, the most remarkable band at 1117 cm^−1^ was typical for G lignin units. Besides, the band at 1462 cm^−1^ is approximate to the reference band at 1514 cm^−1^; the peak at 1215 cm^−1^ was higher than that at 1270 cm^−1^; in the lower wavenumber range, there was a single band observed at 828 cm^−1^. Based on these features, the MWL can be identified as the type GS 3. However, as for the MIBK-lignin, some differences should be noticed in the fingerprint region. The reference band at 1514 cm^−1^ shows more superior to the peak at 1462 cm^−1^, and in the lower wavenumber region, the double peaks which have been circled at 824 cm^−1^ and 850 cm^−1^ can be recognized. Moreover, shoulder peaks (have been circled) can be observed attaching to the bands at 1117 cm^−1^ and 1328 cm^−1^, respectively, which were related to the H units of lignin. According to the statements above, the variation on FT-IR spectra indicated the chemical structures of MIBK-lignin were converted remarkably, particularly the H-type might be formed. However, the details should be identified by further analysis. 

### 3.4. 2D HSQC NMR Analysis

**Figure 3:**
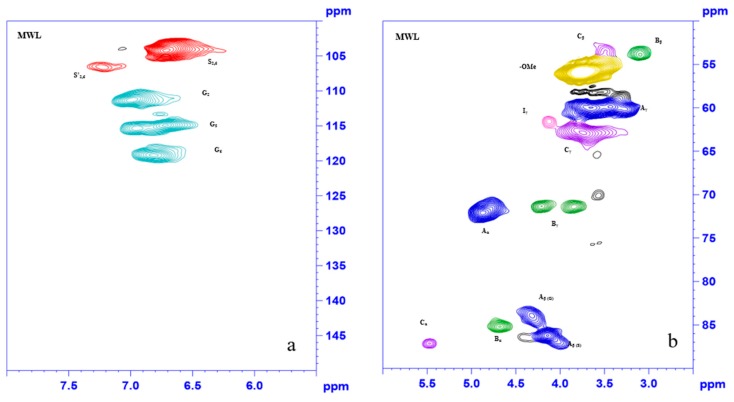

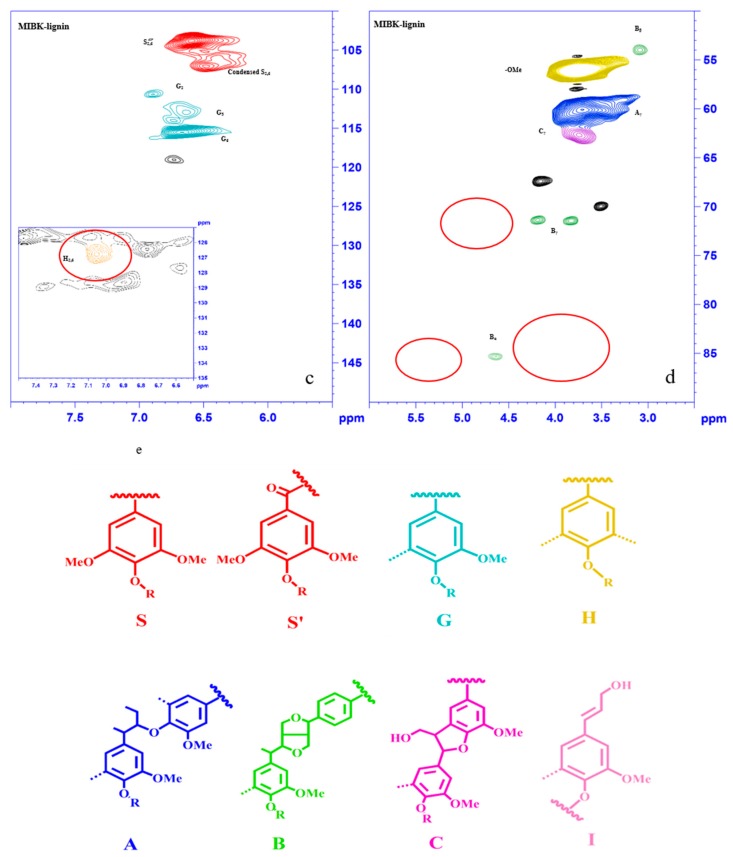
2D HSQC NMR spectra of MWL and MIBK-lignin. (**a**) the cross-peaks from aromatic ring region of MWL (**b**) the cross-peaks from side-chain region of MWL (**c**) the cross-peaks from aromatic ring region of MIBK-lignin (**d**) the cross-peaks from side-chain region of MIBK-lignin (**e**) the chemical structures of lignin units.

2D HSQC NMR is one of the most important characterization methods for comprehending lignin chemical structures. MIBK-lignin was analyzed, compared with MWL, and the spectra of the lignin samples are shown in Figure 3.

The cross-peaks in Figure 3 were marked according to the cross-signal assignments published in the previous research [39]. The aromatic ring region (*δ*_C_/*δ*_H_ 150–100/5.5–8) expressed information about aromatic composition. Peaks from MWL corresponding to S_2,6_ (104.11/6.64), S’_2,6_ (106.59/7.23), G_2_ (111.22/6.93), G_5_ (114.47/6.72), and G_6_ (119.03/6.80) were clearly observed. The condensed S type lignin peak was observed in MIBK-lignin, and the intensity of the G_2_ cross-signal was reduced sharply. Moreover, a weak signal assigned to H_2,6_ (127.11/7.17) (has been circled) appeared. These variations indicated that the S units condensed during the pretreatment and the type of lignin units changed, specifically, a new lignin unit, H unit was formed, which was coincident with the conclusion from FT-IR analysis. The region (*δ*_C_/*δ*_H_ 90–50/6–2.5) corresponding to the side-chain of the lignin, revealed the variation of lignin interunit linkages during the pretreatment. Figure 3b showed the three main lignin interunit linkages, aryl ether linkage (A), resinols (B), and phenylcoumarans (C) in original *Eucalyptus* sample, and these cross-signals were distinguished at *δ*_C_/*δ*_H_ 71.8142/4.8280 (A_α_), 85.1310/4.6798 (B_α_), and 87.1398/5.4720 (C_α_), respectively. However, in the MIBK-lignin spectra, the signals attributing to aryl ether linkage and phenylcoumarans at *δ*_C_/*δ*_H_ 71.8142/4.8280 and 87.1398/5.4720 (have been circled) were disappeared, while the peak at *δ*_C_/*δ*_H_ 85.1310/4.6798 (B_α_) were retained. The percentage of the several classes of lignin interunit linkages were also calculated in detail according to the previous study [5] and showed in Table 3. According to the table, most lignin units of the MWL samples were linked by aryl ether linkage, accounting for 80.03%, followed by the resinols (15.90%) with a slight of phenylcoumarans (4.06%). After the pretreatment, only the resinols remained. Additionally, S/G ratio increased sharply, which resulted from the severe degradation of G-type lignin. Based on the above, the aryl ether linkage and phenylcoumarans were broken selectively during the pretreatment, causing the significant decrease of molecular weight; however, the resinols were more resistant. G-type lignin is more sensitive than the S-type, and after the pretreatment, H-type lignin was formed, indicating the occurrence of demethoxylation reaction on high temperature [25]. The results were coincident with the FT-IR and GPC analysis. 

### 3.5. Morphological Observations

**Figure 4:**
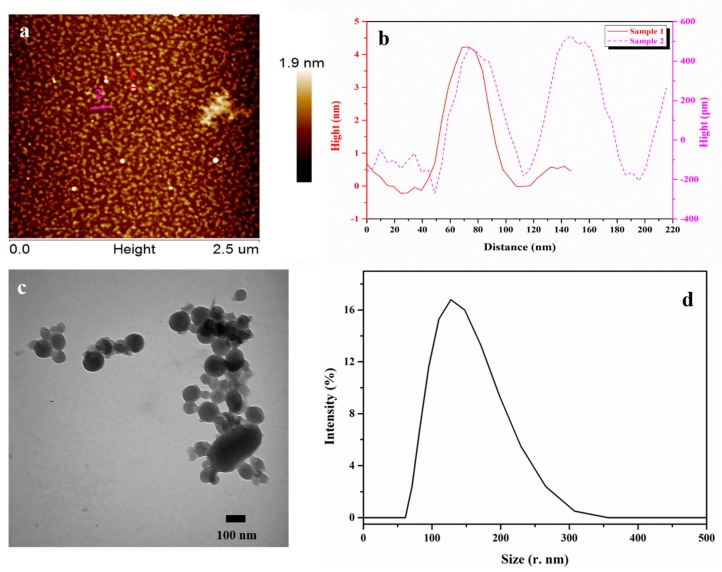
Morphological observation of MIBK-lignin. (**a**) AFM image of MIBK-lignin (**b**) the profiles of samples (**c**) TEM image of MIBK-lignin (**d**) particle diameters distribution analyzed by DLS. (r.: diameter)

The MIBK-lignin was identified as nano-particles by atomic force microscopy (AFM), showed in Figure 4. The freeze-dried MIBK-lignin was firstly dispersed in deionized water at a concentration of 2 wt % followed by ten-minute ultrasound treatment. The lignin nano-particles in upper suspension liquid was observed on a clean mica surface. Due to precipitation, filtration and freeze-drying, the agglomerations of the lignin nano-particles can be observed. Despite this, plenty of homogeneous lignin nano-particles were still clearly seen in Figure 4a. As shown in Figure 4b, the profiles of samples 1 and 2, respectively, corresponding to lines 1 and 2 in Figure 4a precisely described the particle diameters. The diameter of the lighter single particle named 1 was 69 nm, and the darker double particles were 49 and 59 nm, respectively. The similar diameters between the two samples indicated that both the lighter and the dark particles are shown in the image were lignin nano-particles (our product), only they are not in the same layer, consequently showing the difference in height. From the TEM image (Figure 4c), the nano-particles can be observed more clearly, appearing as spheres with diameters about 100 nm. However, some agglomerations still can be seen. Dynamic light scattering (DLS) was applied subsequently for the more accurate particle diameters distribution (Figure 4d). The particle diameters ranged from 70.89 to 307.6, with a mode number of 127.5 nm, and the average diameter of the measured samples was 131.8 nm, with the PDI of 0.149. The combined analyses of AFM, TEM, and DLS visually confirmed that a series of homogeneous lignin nano-particles could easily be obtained by the Lewis acid ferric trichloride hydrolysis modified organosolv pretreatment. Based on the results discussed above, our lignin nano-particles which are directly separated from biomass exhibit great potential in producing high functional and valuable materials applying in wide fields, such as the addition for functional membrane and natural rubber composites, or lignin-based nano-capsules for pharmaceutical applications [40,41,42,43]. 

## 4. Conclusions 

This work proposed a simple biorefinery process, particularly to obtain the lignin nano-particles from *Eucalyptus* biomass by the Lewis acid ferric trichloride hydrolysis modified organosolv pretreatment with MIBK. Under the conditions (180 °C, 1 h), 40.13% of the acid-insoluble lignin in original biomass can be obtained with a high purity of 100% and a low molecular weight of 767 (*M*_n_). The obtained lignin sample was also improved in homogeneity and thermostability characterized by GPC and TGA, respectively. According to FT-IR analysis, the chemical structures of the extracted lignin were converted remarkably as compared to MWL, particularly the H-type unit might be formed. 2D NMR spectroscopy analysis further confirmed the conversion in detail. The aryl ether linkage and phenylcoumarans were broken severely; however, the resinols is more resistant. Besides, G-type lignin is more sensitive to degradation, than the S-type, and after the pretreatment, H-type lignin was formed, indicating the occurrence of demethoxylation reaction at high temperature. Moreover, the lignin nano-particles were identified visually by AFM and TEM images. The DLS was also applied for the more accurate particle diameters distribution and showed that the average diameter of the measured samples was 131.8 nm, with the PDI of 0.149. Results indicate that MIBK-lignin nano-particles have great potential in producing high functional and valuable materials with wide field applications.

## Figures and Tables

**Table 1:** The yields, purity, molecular weight, and polydispersity index (PDI) from MWL and MIBK-lignin.

Component	Yield ^a^ (%)	Purity ^b^ (%)	*M*_n_ (g/mol)	*M*_w_ (g/mol)	PDI
MIBK-lignin	40.31	100	767	1227	1.60
MWL	8.53	96.47	1940	3417	1.76

^a^: the yield was calculated based on the AIL in the original biomass sample. ^b^: the purity was obtained by subtracting the oligosaccharide fraction in the hydrolysate from composition analysis.

**Table 2:** The molecular weights of extracted lignin reported in the literature.

	Conditions	*M*_w_ (g/mol)	*M*_n_ (g/mol)	References
ChCl-lactic acid	140 °C, 8 h	1517	1329	[34]
*p*-TsOH solution	80 °C, 20 min	5400	2100	[35]
ChCl-lactic acid	145 °C, 9 h	1340	- ^a^	[30]
ChCl-oxalic acid	130 °C, 15 min (MW)	2373	208	[36]
ChCl-formic acid	150 °C, 15 min (MW)	5691	1647	[36]
Ethanol/water- FeCl_3_	180 °C, 1.5 h	1642	956	[37]
MIBK-water-FeCl_3_	180 °C, 1 h	1227	767	This work

^a^: “-” means not detected.

**Table 3:** Percentage of lignin interunit linkages and units in the samples.

Samples	Aryl Ether (A)	Resinols (B)	Phenylcoumarans (C)	S/G Ratio
**MWL**	80.03	15.90	4.06	1.13
**MIBK-lignin**	- ^a^	100	-	9.11

^a^: “-” means not detected.
